# Allogeneic hematopoietic cell transplantation for acute myeloid leukemia with *BCR::ABL1* fusion

**DOI:** 10.1002/jha2.877

**Published:** 2024-03-30

**Authors:** Shohei Mizuno, Akiyoshi Takami, Koji Kawamura, Kaito Harada, Masuko Masayoshi, Shingo Yano, Ayumu Ito, Yukiyasu Ozawa, Fumihiko Ouchi, Takashi Ashida, Yuichiro Nawa, Tatsuo Ichinohe, Takahiro Fukuda, Yoshiko Atsuta, Masamitsu Yanada

**Affiliations:** ^1^ Department of Internal Medicine Division of Hematology Aichi Medical University of School of Medicine Nagakute Japan; ^2^ Department of Hematology Tottori University Hospital Yonago Japan; ^3^ Department of Hematology and Oncology Tokai University School of Medicine Isehara Japan; ^4^ Department of Hematopoietic Cell Therapy Niigata University Medical and Dental Hospital Niigata Japan; ^5^ Division of Clinical Oncology and Hematology The Jikei University School of Medicine Tokyo Japan; ^6^ Department of Hematopoietic Stem Cell Transplantation National Cancer Center Hospital Tokyo Japan; ^7^ Department of Hematology Japanese Red Cross Aichi Medical Center Nagoya Daiichi Hospital Nagoya Japan; ^8^ Hematology Division Tokyo Metropolitan Cancer and Infectious Diseases Center Komagome Hospital Tokyo Japan; ^9^ Division of Hematology and Rheumatology Kindai University Hospital Osakasayama Japan; ^10^ Division of Hematology Ehime Prefectural Central Hospital Ehime Japan; ^11^ Department of Hematology and Oncology Research Institute for Radiation Biology and Medicine Hiroshima University Hiroshima Japan; ^12^ Japanese Data Center for Hematopoietic Cell Transplantation Nagakute Japan; ^13^ Department of Registry Science for Transplant and Cellular Therapy Aichi Medical University School of Medicine Nagakute Japan; ^14^ Department of Hematology and Oncology Nagoya City University East Medical Center Nagoya Japan

**Keywords:** acute myeloid leukemia, allogeneic hematopoietic cell transplantation, BCR::ABL1

## Abstract

*BCR::ABL1* fusion is found in < 1% of de novo acute myeloid leukemia (AML) cases and confers a poor prognosis. This Japanese nationwide survey analyzed patients with AML (*n* = 22) and mixed phenotype acute leukemia (MPAL) (*n* = 10) with t(9;22) or *BCR::ABL1* who underwent allogeneic hematopoietic cell transplantation (allo‐HCT) between 2002 and 2018. The 3‐year overall survival (OS) rates were 81.3% and 56.0%, respectively (*p *= 0.15), and leukemia‐free survival (LFS) rates were 76.2% and 42.0%, respectively (*p* = 0.10) in patients with AML and MPAL. The relapse rates were 9.5% and 14.0% (*p* = 0.93), and the non‐relapse mortality (NRM) rates were 14.3% and 44.0%, respectively (*p* = 0.10) in patients with AML and MPAL. One in 17 patients with AML, with pre‐transplant tyrosine kinase inhibitors (TKI), and three in five patients with AML, without pre‐transplant TKI, did not achieve complete remission (CR) before allo‐HCT (*p* = 0.024). Among the 20 patients with known disease status after allo‐HCT, 95.0% were in hematological or molecular CR. None of the four patients who received post‐transplant TKI for prophylaxis or measurable residual disease relapse experienced hematological relapse. In conclusion, our results suggest that pre‐transplant TKI could improve disease status before allo‐HCT. Moreover, allo‐HCT resulted in high OS, high LFS, low relapse, and low NRM rates in patients with AML with *BCR::ABL1*.

## 1 INTRODUCTION

*BCR::ABL1* fusion is a genetic hallmark of chronic myeloid leukemia (CML) and is found in approximately 20%–50% of adult patients with acute lymphoblastic leukemia (ALL) [1]. Acute myeloid leukemia (AML) with *BCR::ABL1* fusion occurs only in < 1% of de novo patients with AML [2, 3]. This is a rare phenomenon and is distinct from the blastic transformation of CML and mixed phenotype acute leukemia (MPAL) with *BCR::ABL1* fusion. The outcome of patients with AML with *BCR::ABL1* receiving standard chemotherapy without allogeneic hematopoietic cell transplantation (allo‐HCT) was poor in the non‐tyrosine kinase inhibitor (TKI) era [4, 5]. According to the European LeukemiaNet (ELN) risk stratification and National Comprehensive Cancer Network guidelines, AML with *BCR::ABL1* fusion is categorized as an adverse risk [6, 7]; therefore, patients should consider allo‐HCT if eligible [8]. However, data on patients who have undergone allo‐HCT are limited. A study reported that the 2‐year overall survival (OS) (68.0%; 95% confidence interval [CI]: 42.1%–84.2%) in patients with AML with *BCR::ABL1* who underwent allo‐HCT is better than that in those with MPAL with *BCR::ABL1* (43.3%; 95% CI: 29.5%–56.3%) [9]. Furthermore, a previous study showed that the 3‐year OS after allo‐HCT was similar between patients with AML including intermediate cytogenetic risk and AML with *BCR::ABL1* fusion (62.7%; 95% CI: 61.0%–64.3% and 73.3%; 95% CI: 51.5%–86.4%, *p* = 0.42); however, it differed significantly between patients with AML including poor cytogenetic risk and AML with *BCR::ABL1* fusion (49.7%; 95% CI: 45.9%–53.4% and 73.3%; 95% CI: 51.5%–86.4%, *p *= 0.049) [10]. Thus, allo‐HCT may improve the prognosis of patients with *BCR::ABL1* fusion. However, only a few studies have evaluated the effect of TKI treatment before and after allo‐HCT, measurable residual disease (MRD) status, and additional cytogenetic abnormalities influencing transplant outcomes in patients with AML with *BCR::ABL1*. Therefore, we conducted a comprehensive survey of patients with AML with *BCR::ABL1* using nationwide registry data and additional questionnaires to clarify how TKI treatment before and after allo‐HCT, MRD status, *BCR::ABL1* subtype, and additional cytogenetic abnormalities influence transplant outcomes.

## 2 MATERIALS AND METHODS

### 2.1 Patient

Clinical data of allo‐HCT recipients were collected from the Transplant Registry Unified Management Program (TRUMP) of the Japanese Society for Transplantation and Cellular Therapy and the Japanese Data Center for Hematopoietic Cell Transplantation [11, 12]. Patients aged ≥ 16 years with AML and MPAL with t(9;22) or *BCR::ABL1*, who underwent allo‐HCT between 2002 and 2018, were included in this study; this timeframe was chosen because imatinib mesylate was first approved for use in Japan in December 2001. Additional questionnaires from each participating center were used. Further clinical information was collected, including detailed characteristics of patients with AML and MPAL with *BCR::ABL1* fusion, treatment with TKI before and after allo‐HCT, and transplant outcomes.

### 2.2 Endpoints and definitions

In this study, the primary endpoint was OS, and the secondary endpoints were leukemia‐free survival (LFS), relapse, and non‐relapse mortality (NRM). Acute and chronic graft‐versus‐host disease (GVHD) were evaluated according to standard criteria [13, 14]. For chronic GVHD analysis, only patients who survived 100 days post‐transplantation without relapse were included. MRD was measured at each institution using qualitative or quantitative polymerase chain reaction of p210 *BCR::ABL1* or p190 *BCR::ABL1* fusion transcripts in the bone marrow. Hematological complete remission (CR) was defined as < 5% blasts in the bone marrow without MRD evaluation, no leukemic blasts in the peripheral blood or extramedullary sites, and no blood count recovery. Molecular CR was defined as MRD‐negative hematological CR. Relapse was defined as the loss of hematological CR in patients who once achieved hematological CR. The Eastern Cooperative Oncology Group performance status [15], HCT‐specific comorbidity index [16], and conditioning intensity [17, 18] were classified according to the published criteria. An alternative donor type was defined as bone marrow or peripheral blood stem cells harvested from human leukocyte antigen‐mismatched donors or cord blood. MPAL with *BCR::ABL1* was determined according to consensus criteria [2]. According to ELN guidelines, myeloid lineage was defined as myeloperoxidase (flow cytometry, immunohistochemistry, or cytochemistry) or monocytic differentiation (at least two of the following: non‐specific esterase cytochemistry, CD11c, CD14, CD64, and lysozyme). T‐lineage was defined as strong cytoplasmic CD3 (with antibodies to CD3 ε chain) or surface CD3. B‐lineage was defined as strong CD19 with at least one of the following strongly expressed: cytoplasmic CD79a, cCD22, or CD10; alternatively, it could be characterized by weak CD19 with at least two of the following strongly expressed: CD79a, cCD22, or CD10 [2]. AML with *BCR::ABL1* was defined as AML with t(9;22)(q34.1;q11.2) or *BCR::ABL1* fusion transcripts, excluding MPAL or the blast transformation of CML.

### 2.3 Statistical analyses

The probability of OS and LFS was calculated using the Kaplan‐Meier method and compared between groups using the log‐rank test. The incidences of relapse, NRM, and GVHD were calculated using cumulative incidence analysis and compared between the groups using Gray's test. In addition, we examined the treatment response before and after allo‐HCT and the impact of each variable on transplantation outcomes in patients with AML with *BCR::ABL1*. All statistical analyses were performed using EZR (Jichi Medical University Saitama Medical Center) [19], which is a graphical user interface for the R software program (version 2.7‐1) (The R Foundation for Statistical Computing).

## 3 RESULTS

### 3.1 Patient characteristics

Ninety‐five cases were extracted from the database, and additional questionnaires were requested from the corresponding institutions. Additional questionnaires were received from 44 cases, of which 12 were not eligible for this study. Finally, 32 patients were enrolled, including 22 and 10 patients who had AML with *BCR::ABL1* and MPAL with *BCR::ABL1*, respectively. The patients’ median (interquartile range) ages were 47 (40.25–58.0) and 43.50 (37.00–52.50) years, respectively, with a median (interquartile range) follow‐up time of 2232.00 (1197.00–2610.50) and 436.50 (146.75–920.50) days, respectively. The patient characteristics are summarized in Table 1. The AML with *BCR::ABL1* group comprised three (13.6%) patients with inv(16) and four (18.2%) with non‐CR at transplantation; however, the MPAL with *BCR::ABL1* group had no such patients. The number of patients treated with TKI before and after transplantation was 17 (77.3%) and five (25.0%) in the AML with *BCR::ABL1* group and seven (77.8%) and one (16.7%) in the MPAL with *BCR::ABL1* group, respectively. Three (60.0%) of the five patients not treated with TKI before allo‐HCT and only one (5.9%) of the 17 patients treated with TKI had non‐CR at transplantation in the AML with *BCR::ABL1* group (*p* = 0.024).

**TABLE 1:** Patient characteristics.

Variables	AML with *BCR::ABL1* (*N* = 22)	MPAL with *BCR::ABL1* (*N* = 10)	*p‐*Value
Age at transplantation, median (IQR), years	47.00 (40.25–58.00)	43.50 (37.00–52.50)	
Age at transplantation, *N* (%)			0.47
< 50 years	12 (54.5)	7 (70.0)	
≥ 50 years	10 (45.5)	3 (30.0)	
Sex, *N* (%)			1.00
Women	10 (45.5)	5 (50.0)	
Men	12 (54.5)	5 (50.0)	
Performance status at transplantation, *N* (%)			
0	21 (100.0)	10 (100.0)	
Missing	1	0	
HCT‐CI, *N* (%)			0.68
0	10 (66.7)	5 (55.6)	
≥ 1	5 (33.3)	4 (44.4)	
Missing	7	1	
*BCR::ABL1* subtype, *N* (%)			0.67
p210	10 (55.6)	3 (42.9)	
p190	8 (44.4)	4 (57.1)	
Missing	4	3	
Additional cytogenetic abnormalities, *N* (%)			0.63
None	10 (45.5)	4 (44.4)	
inv(16)	3 (13.6)	0 (0.0)	
Others	9 (40.9)	5 (55.6)	
Missing	0	1	
Disease status at transplantation, *N* (%)			0.61
Molecular CR	8 (36.4)	5 (55.6)	
Hematological CR	4 (18.2)	2 (22.2)	
Hematological CR with MRD‐positive	6 (27.3)	2 (22.2)	
Non‐CR	4 (18.2)	0 (0.0)	
Missing	0	1	
Donor type, *N* (%)			0.70
HLA‐matched related/unrelated donor	9 (40.9)	3 (30.0)	
Alternative donor	13 (59.1)	7 (70.0)	
HLA 1 alle mismatched related donor	1	0	
HLA‐haploidentical related donor	4	0	
HLA‐mismatched unrelated donor	6	1	
Cord blood	2	6	
Conditioning regimen, *N* (%)			0.44
MAC	14 (63.6)	8 (80.0)	
BU/CY‐based	2	1	
CY/TBI‐based	7	6	
Other TBI‐based	1	0	
Other non‐TBI‐based	4	1	
RIC	8 (36.4)	2 (20.0)	
FLU/BU‐based	4	1	
FLU/MEL‐based	4	0	
Other	0	1	
GVHD prophylaxis, *N* (%)			0.41
Cyclosporine‐based	5 (22.7)	4 (40.0)	
Tacrolimus‐based	17 (77.3)	6 (60.0)	
TKI before transplantation, *N* (%)			1.00
No	5 (22.7)	2 (22.2)	
Yes	17 (77.3)	7 (77.8)	
Missing	0	1	
TKI after transplantation, *N* (%)			1.00
No	15 (75.0)	5 (83.3)	
Yes	5 (25.0)	1 (16.7)	
Hematological relapse	1	1	
MRD relapse	1	0	
Prophylaxis	3	0	
Missing	2	4	
Year of transplantation, median (range)	2009 (2002–2017)	2014 (2004–2018)	
Year of transplantation, *N* (%)			0.45
2002–2009	11 (50.0)	3 (30.0)	
2010–2018	11 (50.0)	7 (70.0)	
Time from diagnosis to transplantation, median (IQR), days	196.50 (136.25–240.50)	138.00 (131.75–158.25)	
Time from diagnosis to transplantation, *N* (%)			0.06
< 6 months	9 (40.9)	8 (80.0)	
≥ 6 months	13 (59.1)	2 (20.0)	

Abbreviations: AML, acute myeloid leukemia; BU, busulfan; CR, complete remission; CY, cyclophosphamide; HCT‐CI, hematopoietic cell transplantation‐specific comorbidity Index; FLU, fludarabine; GVHD, graft‐versus‐host disease; HLA, human leukocyte antigen; IQR, interquartile range; MAC, myeloablative conditioning; MEL, melphalan; MPAL, mixed phenotype acute leukemia; MRD, measurable residual disease; RIC, reduced‐intensity conditioning; TBI, total body irradiation; TKI, tyrosine kinase inhibitor.

### 3.2 Transplant outcomes in patients with AML and MPAL with *BCR::ABL1*

**FIGURE 1:**
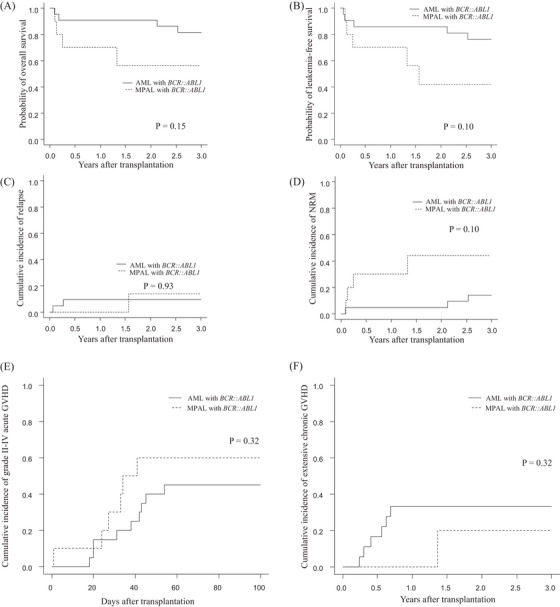
Transplantation outcomes in the AML with *BCR::ABL1* and MPAL with *BCR::ABL1* groups. (A) Overall survival. (B) Leukemia‐free survival. (C) Cumulative incidence of relapse. (D) Cumulative incidence of non‐relapse mortality. (E) Cumulative incidence of grades II–IV acute graft‐versus‐host disease. (F) Cumulative incidence of extensive chronic graft‐versus‐host disease. AML, acute myeloid leukemia; MPAL, mixed phenotype acute leukemia.

The 3‐year probabilities of OS were 81.3% (95% CI: 57.6%–92.6%) and 56.0% (95% CI: 19.7%–81.3%) in the AML with *BCR::ABL1* and MPAL with *BCR::ABL1* groups, respectively (*p* = 0.15) (Figure 1A). The 3‐year LFS probabilities were 76.2% (95% CI: 51.9%–89.3%) and 42.0% (95% CI: 10.7%–71.5%), respectively (*p *= 0.10) (Figure 1B). The 3‐year cumulative incidences of relapse were 9.5% (95% CI: 1.5%–26.6%) and 14.0% (95% CI: 0.40%–50.3%), respectively (*p* = 0.93) (Figure 1C). The 3‐year cumulative incidences of NRM were 14.3% (95% CI: 3.4%–32.7%) and 44.0% (95% CI: 10.5%–74.2%), respectively (*p* = 0.10) (Figure 1D). The cumulative incidences of grades II–IV acute GVHD on day 100 were 45.0% (95% CI: 22.3%–65.4%) and 60.0% (95% CI: 21.8%–84.3%), respectively (*p* = 0.32) and those of extensive chronic GVHD at 3 years were 33.3% (95% CI: 13.1%–55.2%) and 20.0% (95% CI: 0.40%–63.2%), respectively (*p* = 0.32) (Figure 1E,F). Causes of death were two, one, one, one, and one cases of bacterial infection, renal failure, acute respiratory distress syndrome, suicide, and unknown sudden death, respectively, in the AML with *BCR::ABL1* group and included two, one, and one cases of bacterial infection, acute GVHD, and acute hepatitis, respectively, in the MPAL with *BCR::ABL1* group (Table S1). We focused on the NRM of the transplant source because cord blood accounted for 60% of all transplants in the MPAL with the *BCR::ABL1* group. The 3‐year cumulative incidences of NRM were 40.0% (95% CI: 7.0%–73.0%) in cord blood and 17.9% (95% CI: 5.3%–36.3%) in bone marrow and peripheral blood, respectively (*p* = 0.28).

### 3.3 Treatment response before and after allo‐HCT and subgroup analyses in patients with AML with *BCR::ABL1*

**FIGURE 2:**
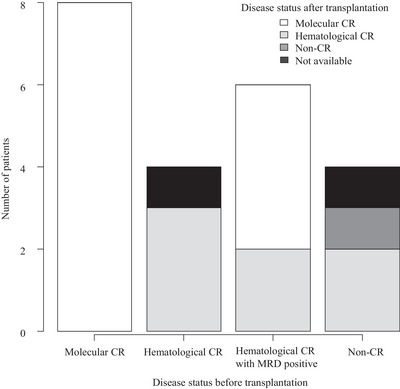
Disease status before and after allo‐HCT in AML with *BCR::ABL1* group. AML, acute myeloid leukemia; allo‐HCT, allogeneic hematopoietic cell transplantation.

We then focused on the characteristics and transplant outcomes in patients with AML with *BCR::ABL1*, comparing disease status before and after allo‐HCT. Among the 20 patients with known disease status after allo‐HCT, 19 (95.0%) had hematological CR or molecular CR (Figure 2). These included four patients with MRD‐positive hematological CR and two with non‐CR before allo‐HCT. We evaluated the prognostic impact of each variable on the transplant outcomes. In a univariate analysis, *BCR::ABL1* subtype, additional cytogenetic abnormalities, and treatment with TKI before and after allo‐HCT were not significantly associated with OS, relapse, and NRM rate (all *p* > 0.05) (Table 2). However, AML with both *BCR::ABL1* and inv(16) group had no relapse or NRM. The disease status before allo‐HCT was significantly associated with OS (*p* = 0.0051) and LFS (*p *= 0.0023) but not with relapse (*p* = 0.52) and NRM (*p* = 0.27). After allo‐HCT, five (22.7%) patients were treated with TKI; among them, three who were treated with TKI for prophylaxis and one for MRD relapse experienced no hematological relapse after the treatment.

**TABLE 2:** Univariable analyses of overall survival, relapse, and non‐relapse mortality rates after 3 years in acute myeloid leukemia (AML) with *BCR::ABL1* group.

	Overall survival		Leukemia‐free survival		Relapse		Non‐relapse mortality	
Variables	% (95% CI)	*p‐*Value	% (95% CI)	*p‐*Value	% (95% CI)	*p‐*Value	% (95% CI)	*p‐*Value
Age at transplantation		0.18		0.066		0.86		0.053
< 50 years	92 (54–99)		92 (54–99)		8 (0–32)		0	
≥ 50 years	68 (29–88)		56 (20–81)		11 (1–41)		33 (7–64)	
Sex		0.53		0.95		0.13		0.19
Women	80 (41–95)		70 (33–89)		20 (3–49)		10 (0–38)	
Men	82 (45–95)		82 (45–95)		0		18 (3–46)	
HCT‐CI		0.16		0.43		0.46		0.19
0	100		89 (43–98)		11 (1–40)		0	
≥ 1	60 (13–88)		60 (13–88)		0		40 (3–79)	
*BCR::ABL1* subtype		0.82		0.76		0.35		0.84
p210	89 (43–98)		78 (37–94)		11 (1–41)		11 (1–41)	
p190	75 (32–93)		75 (32–93)		0		25 (3–58)	
Additional cytogenetic abnormalities		0.43		0.25		0.32		0.65
None	70 (33–89)		60 (25–83)		20 (3–49)		20 (3–49)	
inv(16)	100		NA (NA–NA)		0		0	
Others	67 (5–95)		67 (5–95)		0		33 (0–83)	
Disease status at transplantation		0.0051		0.023		0.52		0.27
Molecular CR	86 (33–98)		71 (26–92)		14 (1–49)		14 (1–50)	
Hematological CR	NA (NA–NA)		NA (NA–NA)		0		0	
Hematological CR with MRD‐positive	NA (NA–NA)		NA (NA–NA)		0		0	
Non‐CR	25 (1–67)		25 (1–67)		25 (0–71)		50 (1–90)	
Donor type		0.55		0.3		0.21		0.83
HLA‐matched Related/unrelated donor	89 (43–98)		89 (43–98)		0		11 (1–41)	
Alternative donor	76 (43–92)		67 (34–86)		17 (2–43)		17 (2–43)	
Conditioning regimen		0.35		0.082		0.04		0.77
MAC	93 (59–99)		93 (59–99)		0		7 (0–29)	
RIC	58 (18–84)		43 (1–73)		29 (3–64)		29 (3–65)	
GVHD prophylaxis		0.66		0.48		0.42		0.85
Cyclosporine‐based	80 (20–97)		80 (20–97)		0		20 (0–62)	
Tacrolimus‐based	82 (54–94)		82 (54–94)		13 (2–34)		13 (2–34)	
TKI before transplantation		0.35		0.52		0.35		0.96
No	60 (13–88)		60 (13–88)		20 (0–62)		20 (0–63)	
Yes	88 (60–97)		81 (53–94)		6 (0–26)		13 (2–34)	
TKI after transplantation		0.50		0.90		0.46		0.69
No	72 (42–89)		71 (41–88)		7 (0–29)		21 (5–46)	
Yes	NA (NA–NA)		80 (20–97)		20 (0–62)		0	
Year of transplantation		0.52		0.94		0.97		0.89
2002–2009	82 (45–95)		82 (45–95)		9 (0–35)		9 (0–35)	
2010–2018	81 (42–95)		70 (33–89)		10 (0–38)		20 (3–49)	
Time from diagnosis to transplantation		0.61		0.39		0.26		0.93
< 6 months	100		NA (NA–NA)		0		0	
≥ 6 months	69 (37–87)		62 (31–82)		15 (2–40)		23 (5–49)	

Abbreviations: AML, acute myeloid leukemia; CR, complete remission; GVHD, graft‐versus‐host disease; HCT‐CI, hematopoietic cell transplantation‐specific comorbidity index; HLA, human leukocyte antigen; MAC, myeloablative conditioning; MRD, measurable residual disease; NA, not available; RIC, reduced‐intensity conditioning; TKI, tyrosine kinase inhibitor.

## 4 DISCUSSION

In this additional nationwide questionnaire‐based study, patients who underwent allo‐HCT for AML with *BCR::ABL1* showed better OS, LFS, and NRM rates than those for MPAL with *BCR::ABL1*. Two reports also observed longer survival in the AML with *BCR::ABL1* group than in the MPAL with *BCR::ABL1* group [9, 20]. AML with *BCR::ABL1* is classified as an adverse‐risk category, and allo‐HCT is the preferred post‐remission therapy for these patients [6, 7]. The European Society for Blood and Marrow Transplantation group reported that the 5‐year OS and relapse rates were 53.8% and 37.0%, respectively, in patients who underwent allo‐HCT for AML with *BCR::ABL1* [8]. The 2‐year OS in the French group [9] and the 5‐year OS in the Korean group [21] were 68.0% and 69.3%, respectively. These studies showed favorable outcomes, and our transplantation results were better. The MPAL with *BCR::ABL1* group was more likely to receive allo‐HCT from cord blood than the AML with *BCR::ABL1* group. In addition, the NRM rate was probably higher in the cord blood group than in the bone marrow and peripheral blood groups. However, our study evaluated a small number of each variable and may not have sufficient statistical power. Therefore, it could not show the significant difference between the transplant sources.

In the AML with *BCR::ABL1* group, the OS of patients with non‐CR compared to that of those with MRD‐positive hematological CR, hematological CR, and molecular CR was unsatisfactory. However, allo‐HCT improved the disease status of patients with MRD‐positive hematological CR and non‐CR. Min et al. reported the 5‐year OS was 100% and 44.4% in the MRD‐negative and MRD‐positive groups, respectively, before allo‐HCT [21]. In addition, MRD‐negative status before allo‐HCT was reported to be associated with improved GVHD‐free relapse‐free survival [8]. However, in our study, the incidence of relapse in the MRD‐positive hematological CR group was favorable and similar to that in the hematological and molecular CR groups. Thus, it remains unclear whether MRD‐positive status affects transplant outcome. These differences may be related to the different MRD levels in each study. The rate of treatment with TKI before allo‐HCT in our study was similar to that reported in a previous study [8] (77.8% and 70.5%, respectively). The MRD‐negative status was previously noted in 12 of the 29 patients treated with TKI before allo‐HCT and in only one of the six patients not treated with TKI [8]. Our study also showed that the rate of non‐CR before allo‐HCT was higher in the non‐TKI‐treated patients than in the TKI‐treated patients. These results suggest the role of TKI in improving the disease status before transplantation. In our study, patients treated with TKI after allo‐HCT for prophylaxis or MRD relapse experienced no hematological relapse. However, because of the small sample size, conclusions regarding the role of TKI after allo‐HCT in patients with AML with *BCR::ABL1* cannot be drawn.

In patients with AML core‐binding factor, additional chromosomal abnormalities do not affect patient outcomes [22]. However, this may not be true when the genetic alteration is *BCR::ABL1*. The most common additional genetic alteration in de novo AML with *BCR::ABL1* is inv(16), with an estimated event rate of 13.5% [23]. Our study included three cases of AML with both *BCR::ABL1* and inv(16). None of these patients experienced relapse or NRM. Similarly, Min et al. showed that all seven patients with t(9;22) and inv(16) were still alive at the end of the study [21]. Moreover, the outcomes of patients with both *BCR::ABL1* and inv(16) were similar between those who underwent allo‐HCT and those who did not [24]. Determining the necessity of allo‐HCT for patients with both *BCR::ABL1* and inv(16) is difficult because of the rarity of this mutation. The p210 transcript was previously found in 30.9%, p190 in 27.8%, both transcripts in 2.4%, and e6a2 in 1.6% of patients with AML with *BCR::ABL1* [23]. Consistent with our cohort, the rates of p210 and p190 were similar except for missing data, and the *BCR::ABL1* subtype did not affect transplant outcomes.

Our study had some limitations. Because of its retrospective nature, there might have been a selection bias. MRD was evaluated at each institution and not in the central laboratory, which might have introduced some bias. Furthermore, we could not assess the concomitant genetic mutation and details of the type, dose, and tolerance of TKIs. Second‐generation TKIs, dasatinib, and nilotinib were approved in Japan in 2009 for newly diagnosed CML. In addition, bosutinib was approved in 2014 for newly diagnosed CML, and ponatinib was approved in 2016 for relapsed and refractory CML and ALL. Therefore, either imatinib, nilotinib, or dasatinib were likely to be prescribed to most patients who received a TKI. A systematic review of case reports [23] evaluated the concomitant genetic mutation in de novo AML with *BCR::ABL1. NPM1* mutation status was evaluated in 15 cases, of which six were positive [23]. Three of these six patients had long‐term survival, one died, and survival data were unavailable for the remaining two [23]. The additional *NPM1* mutation may influence the overall prognosis of patients with AML with *BCR::ABL1*. However, the small number of these patients does not permit a definitive conclusion. Moreover, *FLT3* mutation status was evaluated in 13 patients, of which two were positive in the study [23]. The impact of additional *FLT3* mutations in AML with *BCR::ABL1* remains unclear. Although a prospective randomized trial comparing patients undergoing allo‐HCT with those undergoing chemotherapy alone is the only way to clarify the superiority of allo‐HCT, such a trial is difficult to conduct owing to the rarity of the disease. However, a nationwide registry‐based study such as ours can provide valuable real‐world data.

## 5 CONCLUSION

In the TKI era, pre‐HCT TKI improved the disease status before allo‐HCT, and allo‐HCT provided high OS, high LFS, low relapse, and low NRM rates in patients with AML with *BCR::ABL1*. These results support the benefit of allo‐HCT combined with pre‐HCT TKI treatment for patients with AML with *BCR::ABL1*, which should be validated in prospective studies.

## AUTHOR CONTRIBUTIONS

Shohei Mizuno designed the research, analyzed the data, performed the statistical analysis, and wrote the first draft of the manuscript. Akiyoshi Takami, Koji Kawamura, Kaito Harada, Masuko Masayoshi, Shingo Yano, and Masamitsu Yanada contributed to the critical review of the manuscript. All the other authors contributed to data collection. All authors approved the final version.

## CONFLICT OF INTEREST STATEMENT

The authors declare no conflict of interest.

## FUNDING INFORMATION

This work was supported in part by the Practical Research Project for Allergic Diseases and Immunology (Research Technology of Medical Transplantation) from the Japan Agency for Medical Research and Development (AMED) under Grant Number 18ek0510023h0002.

## ETHICS STATEMENT

This study was approved by the Institutional Review Board of Aichi Medical University.

## PATIENT CONSENT STATEMENT

Informed consent was obtained in writing from each patient.

## CLINICAL TRIAL REGISTRATION

The authors have confirmed clinical trial registration is not needed for this submission.

## Supporting information

Supporting Information

## Data Availability

Datasets from the Transplant Registry Unified Management Program database are not publicly available outside of working groups. Access to these datasets requires permission from the commission. The corresponding author can be contacted at shohei@aichi‐med‐u.ac.jp if the details of the datasets are required.
