# Supplementary material for: Allogeneic hematopoietic cell transplantation for acute myeloid leukemia with BCR::ABL1 fusion
Source: EJHaem. 2024 Mar 30;5(2):369–78. doi: 10.1002/jha2.877 (PMC11020130; doi:10.1002/jha2.877)
Supplement: Supplementary file 1 — Supporting Information [file JHA2-5-369-s001.docx]

| Supplementary table 1. Number of cases of mortality and cause of death | | |
| --- | --- | --- |
|  | AML with *BCR::ABL1* (N = 22) | MPAL with *BCR::ABL1* (N = 10) |
| Mortality | 6 | 4 |
| Bacterial infection | 2 | 2 |
| Acute GVHD | 0 | 1 |
| Acute hepatitis | 0 | 1 |
| Renal failure | 1 | 0 |
| ARDS | 1 | 0 |
| Suicide | 1 | 0 |
| Unknown sudden death | 1 | 0 |
| AML, acute myeloid leukemia; MPAL, mixed phenotype acute leukemia; GVHD, graft-versus-hostdisease; ARDS, acute respiratory distress syndrome | | |
